# Inflammation and Oxidative Stress Induce NGF Secretion by Pulmonary Arterial Cells through a TGF-β1-Dependent Mechanism

**DOI:** 10.3390/cells11182795

**Published:** 2022-09-07

**Authors:** Clément Bouchet, Guillaume Cardouat, Matthieu Douard, Florence Coste, Paul Robillard, Frédéric Delcambre, Thomas Ducret, Jean-François Quignard, Pierre Vacher, Isabelle Baudrimont, Roger Marthan, Patrick Berger, Christelle Guibert, Véronique Freund-Michel

**Affiliations:** 1 Centre de Recherche Cardio-Thoracique de Bordeaux, University Bordeaux, U1045, 33600 Pessac, France; 2 INSERM (Institut National de la Santé Et de la Recherche Médicale), Centre de Recherche Cardio-Thoracique de Bordeaux, U1045, 33600 Pessac, France; 3 IHU Institut de Rythmologie et Modélisation Cardiaque (LIRYC), 33600 Pessac, France; 4 Laboratoire de Pharm-Écologie Cardiovasculaire (LaPEC-EA 4278), Université d’Avignon et des Pays du Vaucluse, 84000 Avignon, France; 5 CHU de Bordeaux, 33000 Bordeaux, France

**Keywords:** nerve growth factor NGF, pulmonary hypertension, oxidative stress, inflammation, transforming growth factor-β1 TGF-β1, pulmonary arterial smooth muscle cells, pulmonary arterial endothelial cells, p38, Smad3

## Abstract

Expression of the nerve growth factor NGF is increased in pulmonary hypertension (PH). We have here studied whether oxidative stress and inflammation, two pathological conditions associated with transforming growth factor-β1 (TGF-β1) in PH, may trigger NGF secretion by pulmonary arterial (PA) cells. Effects of hydrogen peroxide (H_2_O_2_) and interleukin-1β (IL-1β) were investigated ex vivo on rat pulmonary arteries, as well as in vitro on human PA smooth muscle (hPASMC) or endothelial cells (hPAEC). TβRI expression was assessed by Western blotting. NGF PA secretion was assessed by ELISA after TGF-β1 blockade (anti-TGF-β1 siRNA, TGF-β1 blocking antibodies, TβRI kinase, p38 or Smad3 inhibitors). TβRI PA expression was evidenced by Western blotting both ex vivo and in vitro. H_2_O_2_ or IL-1β significantly increased NGF secretion by hPASMC and hPAEC, and this effect was significantly reduced when blocking TGF-β1 expression, binding to TβRI, TβRI activity, or signaling pathways. In conclusion, oxidative stress and inflammation may trigger TGF-β1 secretion by hPASMC and hPAEC. TGF-β1 may then act as an autocrine factor on these cells, increasing NGF secretion via TβRI activation. Since NGF and TGF-β1 are relevant growth factors involved in PA remodeling, such mechanisms may therefore be relevant to PH pathophysiology.

## 1. Introduction

The nerve growth factor NGF belongs to the neurotrophin family and was initially characterized and given its name for its essential role in nerve growth and survival [1]. However, many cells outside the nervous system can secrete NGF, such as inflammatory or structural cells. In addition, NGF effects are not restricted to the nervous system since NGF plays several physiological or pathophysiological roles in various tissues of many organs in the human body [2]. In particular, NGF appears to play a role in the lung, contributing to inflammation and tissue remodeling in several pulmonary inflammatory diseases [3].

In line with these findings, we have recently shown that NGF plays a pathophysiological role in pulmonary hypertension (PH) [4,5]. PH is a life-threatening disease, characterized by elevated pulmonary arterial resistance and pressures, leading to right heart failure and patients’ death [6]. The current available treatments are only symptomatic and do not alter the progression of the disease [7]. Therefore, there is an urgent need for the identification of new therapeutic targets to develop more effective treatments [6,8], and in this context, our results suggest that NGF may be a novel therapeutic target of interest in PH. NGF can trigger proliferation and migration of both pulmonary arterial smooth muscle (PASMC) and endothelial cells (PAEC) and stimulate these cells to secrete pro-inflammatory cytokines such as interleukin-1β (IL-1β) or tumor necrosis factor-α (TNF-α) [5]. In vivo, treatment with anti-NGF blocking antibodies displays both preventive and curative effects in animal models of experimental PH, by significantly decreasing pulmonary arterial remodeling, inflammation, and altered reactivity, which are all hallmarks of PH pathophysiology [5].

In the present work, we were interested in determining the mechanism involved in NGF increased expression in PH. We focused on pulmonary arterial structural cells (namely PASMC and PAEC) as a possible source of increased NGF in pulmonary arteries. Indeed, we have previously shown that both PASMC and PAEC express NGF [5,9]. Inflammation, a critical PH pathological feature [10,11,12], can trigger NGF secretion from different cell types outside the lung, for example, urothelial cells [13], synovial fibroblasts [14], chondrocytes [15], or various cells of the gastrointestinal tract [16,17,18]. Inflammation can also increase NGF expression in the lung, for example in pulmonary fibroblasts [19], bronchial epithelial [20] or smooth muscle cells [21]. In parallel, oxidative stress, another critical PH pathological condition [12,22,23], can enhance NGF secretion from various cell types, for example in the heart [24], the eye [25], or the nervous system [26,27]. We therefore hypothesized that inflammatory and/or oxidative stress conditions may enhance NGF secretion from PASMC and/or PAEC, thus contributing to NGF-increased pulmonary arterial expression in PH. We thus used the pro-inflammatory cytokine IL-1β or hydrogen peroxide (H_2_O_2_) to mimic in vitro inflammatory or oxidative stress conditions, respectively. Human PASMC or PAEC were treated with either IL-1β or H_2_O_2_, and we then evaluated NGF secretion by these cells. We also investigated the mechanism involved in the NGF increased secretion we observed and focused on the role of the transforming growth factor-β1 (TGF-β1), another growth factor whose expression is also increased in PH [28,29,30], and which also plays a pathophysiological role in this disease [31,32,33,34].

## 2. Materials and Methods

### 2.1. Human Pulmonary Arterial Cell Cultures

Pulmonary arteries from control donors were dissected to isolate human PASMC (hPASMC) and PAEC (hPAEC). Further details regarding procedures for human lung sample collection and available donors’ characteristics can be found in the online supplement (supplementary methods and Table S1). hPAEC were cultured in Endothelial Cell Basal Medium^®^ (Promocell, Heidelberg, Germany), which was supplemented with growth factors for cell culture and the DetachKit^®^ for trypsinization (Promocell). Some experiments were also conducted on hPAEC obtained commercially (Promocell). Cell characterization as endothelial cells was done immunocytochemically (expression of CD31 and von Willebrand Factor) (Figure S1a), showing approximately 90% purity. hPASMC were cultured from explants as previously described [5]. Cells were then passaged and cultured in Dulbecco’s Modified Eagle Medium (DMEM, Sigma-Aldrich, Saint-Quentin-Fallavier, France) supplemented with 10% fetal calf serum (ThermoFisher Scientific, Illkirch, France), penicillin (100 units/mL) and streptomycin (100 µg/mL) (both from Sigma-Aldrich) at 37 °C and 5% CO_2_. Cell characterization as smooth muscle cells was done morphologically (typical “hills and valleys” morphology) and immunocytochemically (expression of α-smooth muscle actin, vimentin, and calponin) (Figure S1b), showing approximately 95% purity. Both cell types (hPASMC or hPAEC) were experimentally used at passage 2–6. Further details are provided in the online supplement regarding procedures for cell isolation and characterization.

### 2.2. Cell Treatments

Before experiments, both cell types (hPASMC or hPAEC) were serum-starved for 24 h. Cells were then treated for 24 h in the absence or presence of either IL-1β (0.1–100 ng/mL, Bio-Techne, Lille, France) or H_2_O_2_ (0.1–50 µM, Alfa Aesar, Kandel, Germany). In some experiments, cells were also treated in the absence or presence of TGF-β1 (0.1–10 ng/mL, Bio-Techne). To investigate the role of TGF-β1 and of its TβRI receptor in the effect triggered by IL-1β or H_2_O_2_, these treatments were also applied with or without anti-TGF-β1 blocking antibodies (1 µg/mL, 30 min pre-treatment, Bio-Techne), or with or without an inhibitor of TβRI receptor kinase activity (SB525334; 1 µM, 30 min pre-treatment, Tocris Bioscience, Bristol, United Kingdom). To investigate TβRI-dependent signaling pathways involved, cells were also treated with IL-1β or H_2_O_2_ in the absence or presence of an inhibitor of p38 (SB203580; 2 µM, 45 min pre-treatment, Tocris Bioscience) or of Smad3 (SIS3; 10 µM, 45 min pre-treatment, Tocris Bioscience). In parallel, cells were treated with TGF-β1 (5 ng/mL) for 10, 30, or 60 min to investigate Smad3 and/or p38 phosphorylation. Finally, to confirm that TGF-β1 secretion was upstream of NGF secretion, cells were also pre-treated with anti-NGF blocking antibodies (1 µg/mL, 45 min pre-treatment, Millipore, Molsheim, France) before IL-1β or H_2_O_2_ treatment.

### 2.3. Short Interfering RNA (siRNA) Knockdown Experiments

SiRNA against TGF-β1 (1 nM, Santa Cruz Biotechnology, Dallas, TX, USA) was transfected in hPASMC or in hPAEC using INTERFERin^®^ as the transfection reagent, in accordance with the manufacturer’s instructions (2 µL/well, Polyplus transfection, Illkirch, France). A non-relevant scrambled oligonucleotide (scramble siRNA-A, 1 nM, Santa Cruz Biotechnology) was used as a control in the same conditions, and the effect of INTERFERin^®^ alone was also investigated. Decreased expression of TGF-β1 after transfection of hPASMC or hPAEC with anti-TGF-β1 siRNA was confirmed by enzyme-linked immunosorbent assay (ELISA) experiments 48 h after the transfections. Cells were then serum-starved for 24 h, treated for 24 h in the absence or presence of IL-1β (10 ng/mL) or of H_2_O_2_ (10 µM), and NGF levels in the supernatants of hPASMC or hPAEC were then measured by ELISA.

### 2.4. Preparation of Rat Pulmonary Arteries

For all animal studies, agreement was obtained from the French authorities (number A33-318-3), and experiments conformed to the Declaration of Helsinki conventions for the use and care of animals. Control male Wistar rats (10-week old, Janvier Labs, Le Genest-Saint-Isle, France) were euthanized by sodium pentobarbital overdose (200 mg/kg ip), and intrapulmonary arteries were dissected from the left and right lungs under binocular control, after thoracotomy and exsanguination. After that, the pulmonary arteries were then incubated in DMEM for 24 h (37 °C, 5% CO_2_, 200 µL for 10 mg tissue). For each rat, intrapulmonary arteries dissected from the left lung were used as controls, whereas intrapulmonary arteries dissected from the right lung were incubated with either IL-1β (10 ng/mL) or H_2_O_2_ (10 µM). NGF or TGF-β1 levels were then measured in the pulmonary arterial supernatants by ELISA.

### 2.5. NGF and TGF-β1 Dosages

After treatments of hPASMC, hPAEC or rat intrapulmonary arteries, supernatants were then collected, snap frozen, and stored at −20 °C until analysis. Determination of NGF or TGF-β1 levels were assessed by use of ELISA kits, according to the manufacturer’s instructions (for NGF rat samples: Rat β-NGF ELISA Kit from Millipore; for NGF human samples: NGF Rapid Kit ELISA Human from Biosensis, Thebarton, Australia; and for TGF-β1 rat and human samples: TGF-β1 E_max_ Immunoassay system from Promega, Charbonnières-les-Bains, France). NGF and TGF-β1 levels were normalized to total tissue or cell protein content, the latter being determined by the Lowry method (Bio-Rad, Hercules, CA, USA).

### 2.6. Quantitative Real-Time PCR

Total RNAs were extracted from hPASMC or hPAEC using NucleoZOL^®^, according to the manufacturer’s instructions (Macherey-Nagel, Hoerdt, France). A quantity of 50 ng RNA (for hPAEC) or 100 ng RNA (for hPASMC) were reverse-transcribed using the High Capacity cDNA reverse transcription kit from Applied Biosystems (Waltham, MA, USA). cDNA samples were then analyzed by qPCR using Quantinova SYBR^®^ Green supermix (Qiagen, Hilden, Germany) through the CFX Connect real-time PCR detection system (Bio-Rad). *NGF* primers (Forward 5′-CGTCCGGACCCAATAACAGT-3′ and Reverse 5′-AGTGTGGTTCCGCCTGTATG-3′) were purchased from Sigma-Aldrich. NGF mRNA expression was determined using the comparative 2^-ΔΔCt^ method and normalized to the mRNA expression level of endogenous references by using geometric averages of 2 or 3 internal housekeeping genes (*RPL13A*, primers: Forward 5′-GGGAGCAAGGAAAGGGTCTTA-3′, Reverse 5′-CACCTGCACAATTCTCCGAGT-3′; *GUSB*, primers: Forward 5′-CCATCTGGGTCTGGATCAAAA-3′, Reverse 5′-TGAAATCGGCAAAATTCCAAAT-3′; and/or *RPLPO*, primers: Forward 5′-TCGTGGAAGTGACATCGTCTTT-3′, Reverse 5′-CTGTCTTCCCTGGGCATCA-3′).

### 2.7. Western Blotting

Control rat dissected intrapulmonary arteries were homogenized in ice-cold radio-immunoprecipitation assay (RIPA) lysis buffer supplemented with 1% *v/v* Nonidet P-40, 0.25% sodium deoxycholate, and 10 µL/mL protease inhibitors (all from Sigma-Aldrich). hPASMC or hPAEC were scraped in the same RIPA lysis buffer. Rat and human samples were then further incubated on ice for 30 min. After centrifugation (15,000× *g*, 10 min, 4 °C), total protein concentrations in the supernatants were assessed by the Lowry method (Bio-Rad). Proteins (60 µg for rat samples or 20 µg for human samples) were then separated by 10% acrylamide gel electrophoresis and transferred onto polyvinylidene difluoride (PVDF) membranes. Membranes were then saturated for 1 h at room temperature (with 0.1% TBS-Tween containing either 5% non-fat milk or 5% bovine serum albumin for phosphorylation studies), incubated overnight at 4 °C with the primary antibody (goat anti-TβRI polyclonal antibodies, 0.5 µg/mL, Bio-Techne; rabbit anti-phospho-Smad3 monoclonal antibodies, 1/500, Cell Signaling Technology, Danvers, MA, USA; mouse anti-Smad3 monoclonal antibodies, 1/500, Santa Cruz Biotechnology; mouse anti-phospho-p38 monoclonal antibodies, 1/1000, Cell Signaling Technology; rabbit anti-p38 polyclonal antibodies, 1/1000, Cell Signaling Technology; mouse anti-NGF monoclonal antibodies, 1/500, Santa Cruz Biotechnology), and further incubated 1 h at room temperature with the corresponding secondary antibodies (donkey anti-goat polyclonal antibodies, 1/5000, ThermoFisher Scientific; goat anti-rabbit polyclonal antibodies, 1/5000, Vector laboratories, Newark, NJ, USA; horse anti-mouse polyclonal antibodies, 1/5000, Vector laboratories). Proteins were then detected on the membranes using chemiluminescence visualization, following the manufacturer’s recommendations (Immobilon^TM^ Western, Millipore). Detection of glyceraldehyde-3-phosphate dehydrogenase (GAPDH) with anti-GAPDH rabbit polyclonal antibodies (1/5000, Sigma-Aldrich) was used in parallel for loading controls.

### 2.8. WST-1 Experiments

Cell viability and proliferation were assessed by use of the WST-1 cell proliferation assay (Water Soluble Tetrazolium-1, Roche Diagnostics, Mannheim, Germany) according to the manufacturer’s instructions. Briefly, cells were seeded in 96-well culture plates (5000 cells per well), cultured for 24 h, serum-starved for 24 h and treated for 24 h with IL-1β or H_2_O_2_. Cells were then exposed to WST-1 reagent for 3 h and absorbance was measured immediately at 450 nm by spectrophotometry.

### 2.9. Statistical Analysis

Results are expressed as raw data, as mean ± standard error of the mean (SEM) or as mean ± standard deviation (SD) of *n* independent observations (with *n* being either the number of independent experiments on cultured cells or the number of rats per experiment, as indicated in each figure legend). Multiple comparisons were performed with a one-way ANOVA followed by the Dunn’s test. All data were analyzed using Graphpad PRISM software (v6, Graphpad Software, San Francisco, CA, USA), with *p* < 0.05 considered significant.

## 3. Results

### 3.1. IL-1β and H_2_O_2_ Increase NGF Secretion by Human Pulmonary Arterial Cells

**Figure 1:**
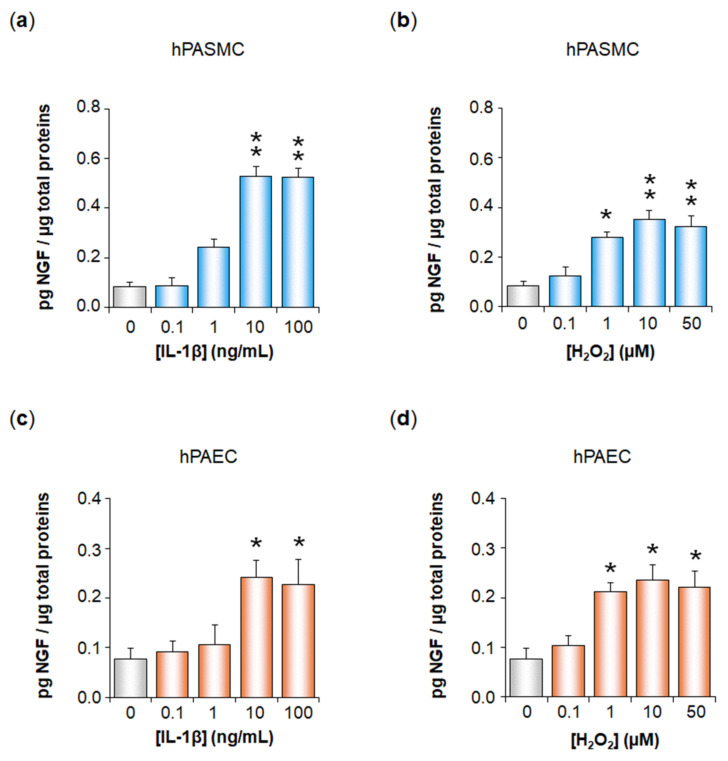
NGF secretion by human pulmonary arterial cells after treatment with IL-1β or H_2_O_2._ NGF secretion by human pulmonary arterial smooth muscle cells (hPASMC, (**a**,**b**)) or by human pulmonary arterial endothelial cells (hPAEC, (**c**,**d**)) was assessed in the absence or presence of interleukin-1β (IL-1β, 0.1–100 ng/mL, 24 h, (**a**,**c**)) or hydrogen peroxide (H_2_O_2_, 0–50 µM, 24 h, (**b**,**d**)). NGF secretion was determined by ELISA (results expressed as pg NGF/µg total proteins). The data represent the means ± SEM of *n* = 5 independent experiments performed in triplicate on cells from three to five control donors. *: *p* < 0.05 and **: *p* < 0.01 versus untreated control cells.

NGF was secreted by hPASMC in basal control conditions and this secretion was significantly increased after cell treatment with IL-1β, with a maximal effect at 10 ng/mL (from 0.09 ± 0.02 to 0.53 ± 0.04 pg NGF/µg total proteins, *p* < 0.01, Figure 1a).

NGF secretion by hPASMC was also significantly increased after cell treatment with H_2_O_2_, with a maximal increase at 10 µM (from 0.09 ± 0.02 to 0.35 ± 0.04 pg NGF/µg total proteins, *p* < 0.01, Figure 1b). Similar results were observed in hPAEC, with NGF basal secretion increased after cell treatment with IL-1β, with a maximal effect at 10 ng/mL (from 0.08 ± 0.02 to 0.24 ± 0.04 pg NGF/µg total proteins, *p* < 0.05, Figure 1c), or after cell treatment with H_2_O_2_, with a maximal increase at 10 µM (from 0.08 ± 0.02 to 0.24 ± 0.03 pg NGF/µg total proteins, *p* < 0.05, Figure 1d).

Experiments were conducted in both hPASMC and hPAEC to confirm that cell viability was not altered, neither by IL-1β nor by H_2_O_2_, whatever the concentration tested (Figure S2). Our results even suggested a dose-dependent increased viability in both hPASMC and hPAEC after treatment with IL-1β, suggesting a proliferative effect of IL-1β on these cells (Figure S2).

NGF increased secretion induced by IL-1β or H_2_O_2_ seems to be related to increased NGF mRNA (Figure S3) and protein (Figure S4) expression in both hPASMC and hPAEC. Indeed, RT-qPCR experiments showed that IL-1β (10 ng/mL) triggered an increase in NGF mRNA relative expression in both hPASMC and hPAEC, with a maximal increase observed after 2 to 4 h of treatment (Figure S3a,c). An increase in NGF mRNA relative expression was also observed in both hPASMC and hPAEC after H_2_O_2_ treatment (10 µM), but with a maximal increase observed later, after 24 h of treatment (Figure S3b,d). In parallel, Western blotting experiments showed an increase in NGF protein expression in both hPASMC and hPAEC after 24 h of either IL-1β (10 ng/mL) or H_2_O_2_ treatment (10 µM) (Figure S4).

### 3.2. TGF-β1 Contributes to IL-1β and H_2_O_2_-Induced NGF Increased Secretion by Human Pul-Monary Arterial Cells, through Activation of Its TβRI Receptor and of Smad3 and/or p38-Dependent Signaling Pathways

**Figure 2:**
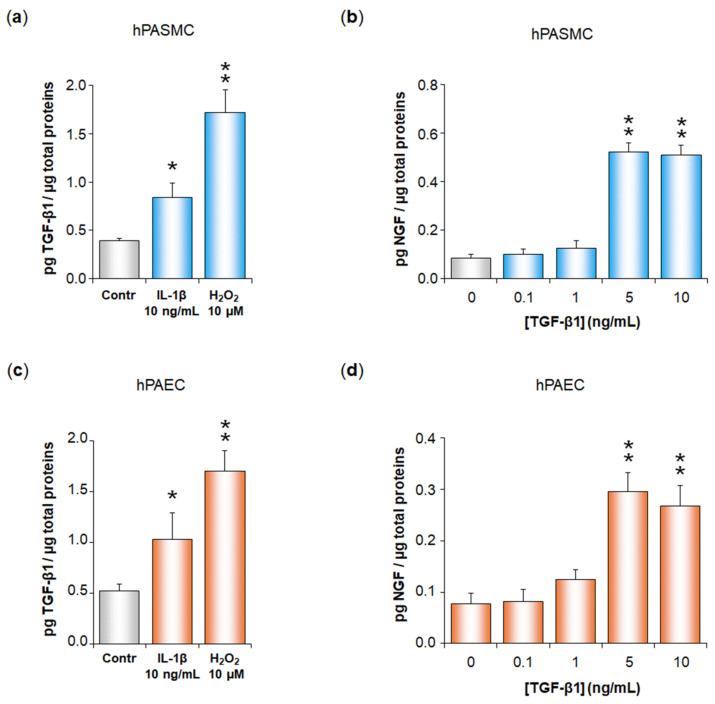
TGF-β1 secretion by human pulmonary arterial cells after treatment with IL-1β or H_2_O_2_, and TGF-β1 ability to trigger NGF secretion by these cells. TGF-β1 secretion by human pulmonary arterial smooth muscle cells (hPASMC, (**a**)) or human pulmonary arterial endothelial cells (hPAEC, (**c**)) was assessed in the absence or presence of interleukin-1β (IL-1β, 10 ng/mL, 24 h) or hydrogen peroxide (H_2_O_2_, 10 µM, 24 h). NGF secretion by (**b**) hPASMC or (**d**) hPAEC was assessed in the absence or presence of TGF-β1 (0.1–10 ng/mL, 24 h). NGF and TGF-β1 secretions were determined by ELISA (results expressed as pg TGF-β1 or pg NGF/µg total proteins). The data represent the means ± SEM of *n* = 5 independent experiments performed in triplicate on cells from three to five control donors. * *p* < 0.05 and ** *p* < 0.01 versus untreated control cells (Contr or 0 ng/mL of TGF-β1).

To determine whether TGF-β plays a role in NGF increased secretion induced by IL-1β or H_2_O_2_ in hPASMC or hPAEC, we first demonstrated that IL-1β (10 ng/mL) or H_2_O_2_ (10 µM) significantly increased TGF-β1 secretion in hPASMC (IL-1β: from 0.39 ± 0.03 to 0.84 ± 0.15 pg TGF-β1/µg total proteins, *p* < 0.05; H_2_O_2_: from 0.39 ± 0.03 to 1.72 ± 0.23 pg TGF-β1/µg total proteins, *p* < 0.01; Figure 2a) as well as in hPAEC (IL-1β: from 0.52 ± 0.07 to 1.03 ± 0.27 pg TGF-β1/µg total proteins, *p* < 0.01; H_2_O_2_: from 0.52 ± 0.07 to 1.70 ± 0.20 pg TGF-β1/µg total proteins, *p* < 0.01; Figure 2c). In parallel, we also showed that cell treatment with exogenous TGF-β1 significantly triggered NGF secretion in both hPASMC and hPAEC, with a maximal effect observed at 5 ng/mL (hPASMC: from 0.09 ± 0.02 to 0.52 ± 0.04 pg NGF/µg total proteins, *p* < 0.01, Figure 2b; hPAEC: from 0.08 ± 0.02 to 0.30 ± 0.04 pg NGF/µg total proteins, *p* < 0.01, Figure 2d).

We conducted further experiments to assess whether TGF-β1 secretion in hPASMC or hPAEC was altered when cells had been pre-treated with anti-NGF blocking antibodies (Figure S4). We showed that NGF blockade did not alter TGF-β1 secretion triggered by IL-1β or H_2_O_2_ neither in hPASMC (Figure S5a,b) nor in hPAEC (Figure S5c,d). These results therefore show that TGF-β1 secretion is upstream of NGF secretion after IL-1β or H_2_O_2_ treatment in hPASMC or hPAEC.

**Figure 3:**
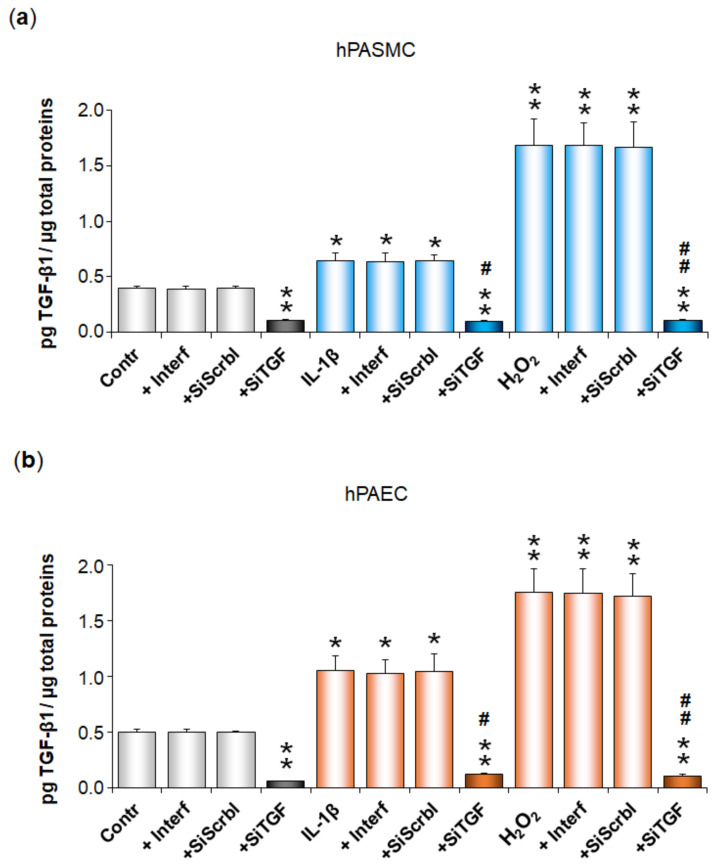
Control of TGF-β1 decreased secretion after transfection of pulmonary arterial cells with an anti TGF-β1 siRNA. TGF-β1 secretion by human pulmonary arterial smooth muscle cells (hPASMC, (**a**)) or human pulmonary arterial endothelial cells (hPAEC, (**b**)) was assessed in the absence or presence of interleukin-1β (IL-1β, 10 ng/mL, 24 h) or hydrogen peroxide (H_2_O_2_, 10 µM, 24 h). Cells were pre-treated for 48 h with an anti-TGF-β1 siRNA (SiTGF, 1 nM). The effect of this anti-TGF-β1 siRNA on TGF-β1 basal secretion was evaluated. Furthermore, the effects of the transfecting agent INTERFERin^®^ (Interf) alone and of a non-relevant scramble siRNA (SiScrbl, 1 nM) on TGF-β1 basal and induced secretions were evaluated. TGF-β1 secretion was determined by ELISA (results expressed as pg TGF-β1/µg total proteins). The data represent the means ± SEM of *n* = 5 independent experiments performed in triplicate on cells from three to five donors. * *p* < 0.05 and ** *p* < 0.01 versus untreated control cells (Contr). ^#^ *p* < 0.05 and ^##^ *p* < 0.01 versus cells treated with either IL-1β or H_2_O_2_.

We then performed experiments in which hPASMC or hPAEC were transfected with an anti-TGF-β1 siRNA. Before further experiments, we first confirmed by ELISA experiments that this transfection decreased TGF-β1 secretion by hPASMC or hPAEC (Figure 3). Indeed, TGF-β1 basal secretion was significantly reduced in hPASMC (from 0.40 ± 0.02 to 0.11 ± 0.01 pg TGF-β1/µg total proteins, *p* < 0.01, i.e., a silencing efficiency of 72%) (Figure 3a) or in hPAEC (from 0.50 ± 0.03 to 0.06 ± 0.01 pg TGF-β1/µg total proteins, *p* < 0.01, i.e., a silencing efficiency of 88%) (Figure 3b). We also showed that TGF-β1 increased secretion triggered by IL-1β or H_2_O_2_ in both hPASMC and hPAEC was also decreased to the same low levels after cell transfection with the anti-TGF-β1 siRNA (Figure 3). Neither the transfecting agent INTERFERin^®^ alone nor the non-relevant scrambled siRNA displayed any significant effect on TGF-β1 secretion, either basal or triggered by IL-1β or H_2_O_2_ in both cell types (Figure 3).

**Figure 4:**
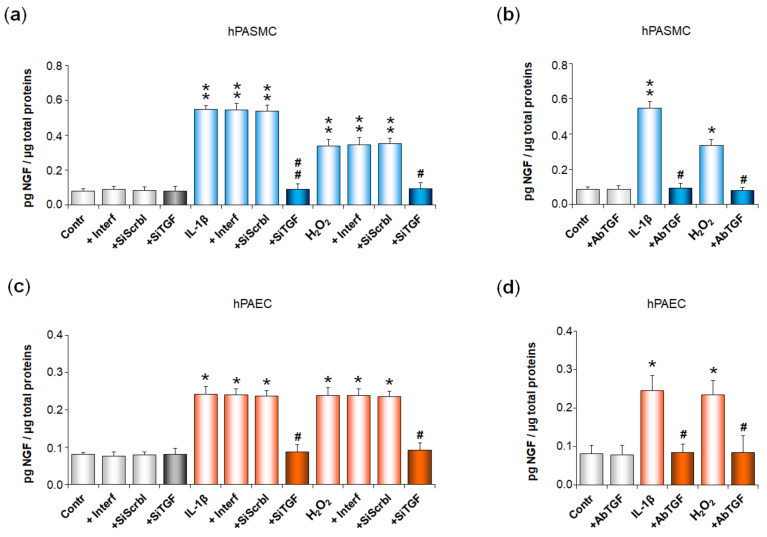
Role of TGF-β1 in IL-1β- or H_2_O_2_-induced increase in NGF secretion by human pulmonary arterial cells. NGF secretion by human pulmonary arterial smooth muscle cells (hPASMC, (**a**,**b**)) or human pulmonary arterial endothelial cells (hPAEC, (**c**,**d**)) was assessed in the absence or presence of interleukin-1β (IL-1β, 10 ng/mL, 24 h) or hydrogen peroxide (H_2_O_2_, 10 µM, 24 h). Involvement of the transforming growth factor-β1 (TGF-β1) was investigated in (**a**) hPASMC or (**c**) hPAEC pre-treated for 48 h with an anti-TGF-β1 siRNA (SiTGF, 1 nM). Effect of this anti-TGF-β1 siRNA on NGF basal secretion was evaluated. Furthermore, the effects of the transfecting agent INTERFERin^®^ (Interf) alone and of a non-relevant scramble siRNA (SiScrbl, 1 nM) on NGF basal and induced secretions were evaluated. TGF-β1 involvement was also investigated through pre-treatment (30 min) of (**b**) hPASMC or (**d**) hPAEC with anti-TGF-β1 blocking antibodies (AbTGF, 1 µg/mL). Effect of these antibodies on NGF basal secretion was evaluated. NGF secretion was determined by ELISA (results expressed as pg NGF/µg total proteins). The data represent the means ± SEM of *n* = 5 independent experiments performed in triplicate on cells from three to five control donors. * *p* < 0.05 and ** *p* < 0.01 versus untreated control cells (Contr). ^#^ *p* < 0.05 and ^##^ *p* < 0.01 versus cells treated with either IL-1β or H_2_O_2_.

We then investigated whether NGF secretion by pulmonary arterial cells was altered after cell transfection with the anti-TGF-β1 siRNA or after cell treatment with anti-TGF-β1 blocking antibodies (Figure 4). In hPASMC, IL-1β- or H_2_O_2_-induced increase in NGF secretion was totally blocked in cells pre-treated either with the anti-TGF-β1 siRNA (*p* < 0.01 or *p* < 0.05, Figure 4a), or with anti-TGF-β1 blocking antibodies (*p* < 0.05, Figure 4b). Similar results were observed in hPAEC pre-treated either with the anti-TGF-β1 siRNA (*p* < 0.05, Figure 4c), or with anti-TGF-β1 blocking antibodies (*p* < 0.05, Figure 4d).

In siRNA experiments, cell treatment with the transfection reagent alone or with a non-relevant scrambled siRNA did not alter NGF basal or induced secretions, neither in hPASMC (Figure 4a), nor in hPAEC (Figure 4c). In addition, cell treatment with the anti-TGF-β1 siRNA (Figure 4a,c) or with anti-TGF-β1 blocking antibodies (Figure 4b,d) did not alter NGF basal secretion in either cell type.

**Figure 5:**
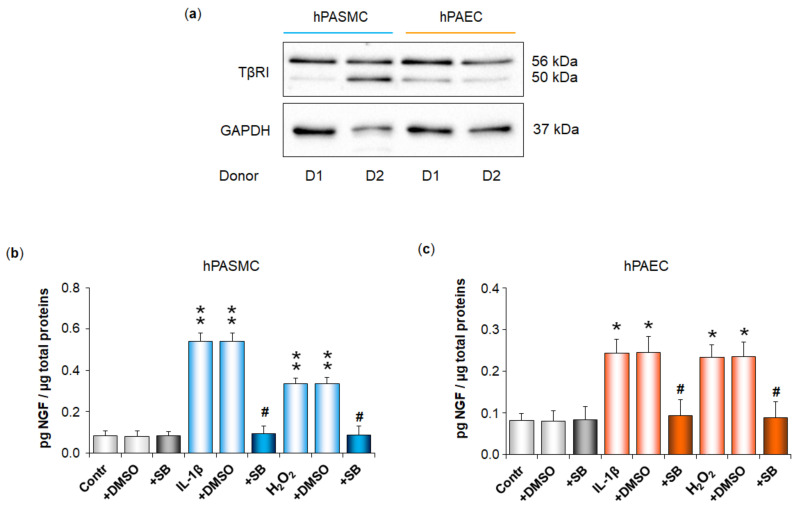
Expression of the TGF-β1 receptor TβRI in human pulmonary arterial cells and its role in IL-1β- or H_2_O_2_-induced increase in NGF secretion by these cells. (**a**) Basal protein expression of TβRI was assessed by Western blotting analysis in human pulmonary arterial smooth muscle cells (hPASMC) or human pulmonary arterial endothelial cells (hPAEC). Immunoblots presented on cells from two control donors (D1 and D2) are representative of experiments conducted on pulmonary arterial cells from *n* = 3–5 control donors, showing identical results. Results are normalized to glyceraldehyde-3-phosphate dehydrogenase (GAPDH). (**b**,**c**) NGF secretion by (**b**) hPASMC or (**c**) hPAEC was assessed in the absence or presence of interleukin-1β (IL-1β, 10 ng/mL, 24 h) or hydrogen peroxide (H_2_O_2_, 10 µM, 24 h). Involvement of TβRI was investigated through pre-treatment (30 min) of (**b**) hPASMC or (**c**) hPAEC with SB525334, an inhibitor of TβRI receptor kinase activity (SB, 1 µM). The effect of this inhibitor on NGF basal secretion was investigated. In addition, the effects of dimethyl sulfoxide (DMSO), the vehicle of the SB compound, on NGF basal and induced secretions were evaluated. NGF secretion was determined by ELISA (results expressed as pg NGF/µg total proteins). The data represent the means ± SEM of *n* = 5 independent experiments performed in triplicate on cells from three to five donors. * *p* < 0.05 and ** *p* < 0.01 versus untreated control cells (Contr). ^#^ *p* < 0.05 versus cells treated with IL-1β or H_2_O_2_.

We then showed that the TGF-β1 receptor TβRI was expressed in both hPASMC and hPAEC (Figure 5a), and that IL-1β- or H_2_O_2_-induced increase in NGF secretion was totally blocked with an inhibitor of TβRI kinase activity (SB525334, 1 µM), in either hPASMC (Figure 5b) or hPAEC (Figure 5c). Cell treatment with the inhibitor vehicle alone (DMSO) did not alter NGF basal or induced secretions, neither in hPASMC (Figure 5b), nor in hPAEC (Figure 5c). In addition, cell treatment with the inhibitor of TβRI kinase activity did not alter NGF basal secretion in either cell type (Figure 5b,c).

**Figure 6:**
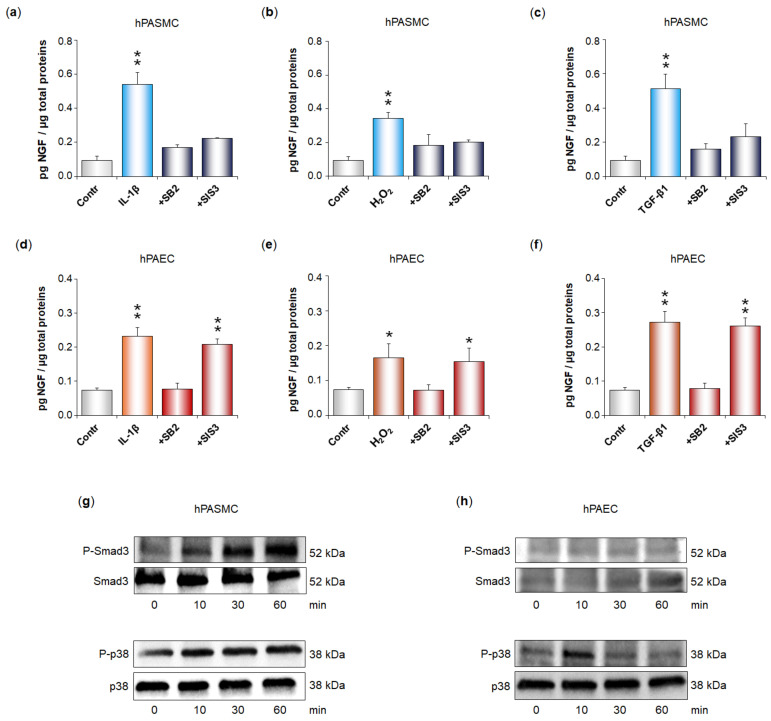
Signaling pathways activated by the TGF-β1 receptor TβRI in human pulmonary arterial cells to participate in the IL-1β- or H_2_O_2_-induced increase in NGF secretion by these cells. (**a**–**f**) NGF secretion by human pulmonary arterial smooth muscle cells (hPASMC, (**a**–**c**)) or human pulmonary arterial endothelial cells (hPAEC, (**d**–**f**)) was assessed in the absence or presence of interleukin-1β (IL-1β, 10 ng/mL, 24 h), hydrogen peroxide (H_2_O_2_, 10 µM, 24 h), or TGF-β1 (5 ng/mL, 24 h). Signaling pathways involved were investigated through cell pre-treatment (45 min) with the p38 inhibitor SB203880 (+ SB2, 2 µM) or with the Smad3 inhibitor SIS3 (+SIS3, 10 µM). NGF secretion was determined by ELISA (results expressed as pg NGF/µg total proteins). The data represent the means ± SEM of *n* = 3 independent experiments performed in triplicate on cells from three control donors. * *p* < 0.05 and ** *p* < 0.01 versus untreated control cells (Contr). (**g,h**) Time-course of TGF-β1-induced phosphorylation of Smad3 and p38 in (**g**) hPASMC or (**h**) hPAEC. Cells were incubated with TGF-β1 (5 ng/mL) for 10–60 min, and Western blotting analysis used anti-phospho-Smad3 or anti-phospho-p38 antibodies, as well as anti-Smad3 and anti-p38 antibodies as controls. The immunoblots presented are representative of experiments conducted on pulmonary arterial cells from *n* = 2–4 control donors, showing identical results.

Finally, we investigated the signaling pathways activated by the TGF-β1 receptor TβRI to trigger NGF secretion in both hPASMC and hPAEC (Figure 6). In hPASMC, IL-1β (Figure 6a), H_2_O_2_ (Figure 6b), and TGF-β1 (Figure 6c) all significantly increased NGF secretion. These effects were totally blocked in the presence of an inhibitor of p38 (SB203580, 2 µM) or in the presence of an inhibitor of Smad3 (SIS3, 10 µM).

Similarly, in hPAEC, IL-1β (Figure 6d), H_2_O_2_ (Figure 6e), and TGF-β1 (Figure 6f) all significantly increased NGF secretion. These effects were totally blocked in the presence of the p38 inhibitor but were still significant in the presence of the Smad3 inhibitor. Accordingly, TGF-β1 (5 ng/mL) induced phosphorylation of both Smad3 and p38 in hPASMC (Figure 6g), whereas it only induced p38 phosphorylation in hPAEC (Figure 6h).

### 3.3. NGF Increased Secretion Triggered by IL-1β in Human Pulmonary Arterial Cells Contributes to IL-1β-Induced Proliferation of These Cells

**Figure 7:**
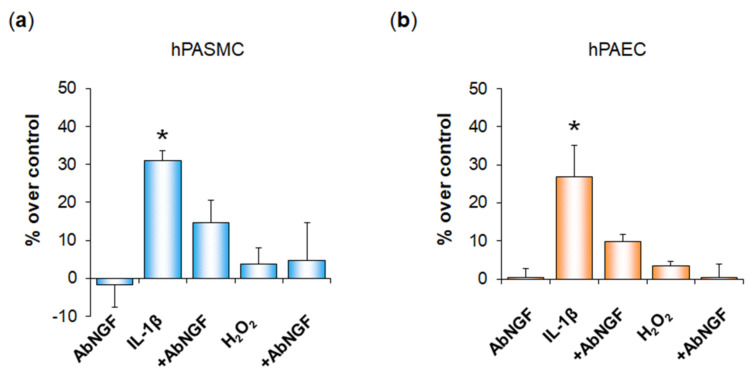
Functional role of NGF secreted by human pulmonary arterial cells after exposure to IL-1β or H_2_O_2._ Proliferation of human pulmonary arterial smooth muscle cells (hPASMC, (**a**)) or of human pulmonary arterial endothelial cells (hPAEC, (**b**)) was assessed in the absence or presence of interleukin-1β (IL 1β, 10 ng/mL, 24 h) or hydrogen peroxide (H_2_O_2_, 10 µM, 24 h). Cell proliferation was assessed by the colorimetric WST-1 cell proliferation assay. Involvement of NGF in IL-1β- or H_2_O_2_-altered proliferation of pulmonary arterial cells was investigated through pre-treatment (45 min) with anti-NGF blocking antibodies (AbNGF, 1 µg/mL). The data are expressed as a percentage of proliferation compared to untreated control cells (% over control) and are presented as the means ± SD of *n* = 3 independent experiments performed in triplicate on cells from three control donors. *: *p* < 0.05 versus untreated control cells.

Assessment of cell viability in our WST-1 experiments suggested that IL-1β triggered proliferation of both hPASMC and hPAEC (see Figure S2). As NGF is a growth factor and has previously been shown to trigger hPASMC and hPAEC proliferation [5], we therefore assessed whether NGF secreted in response to IL-1β may contribute to the IL-1β-dependent proliferation of these cells. We confirmed in additional WST-1 experiments that IL-1β significantly increased hPASMC (Figure 7a) and hPAEC proliferation (Figure 7b) and showed that this effect was blocked when cells were pre-treated with anti-NGF blocking antibodies. In parallel, these additional experiments showed again that H_2_O_2_ did not alter cell viability and demonstrated that pre-treatment with anti-NGF blocking antibodies had no effect on cells treated with H_2_O_2_ (Figure 7). Finally, we also ensured that cell treatment with anti-NGF blocking antibodies alone did not alter cell viability in neither hPASMC (Figure 7a) nor in hPAEC (Figure 7b).

### 3.4. IL-1β and H_2_O_2_ Increase NGF Secretion by Rat Whole Pulmonary Arteries through a Similar Mechanism than in Human Pulmonary Arterial Cells

**Figure 8:**
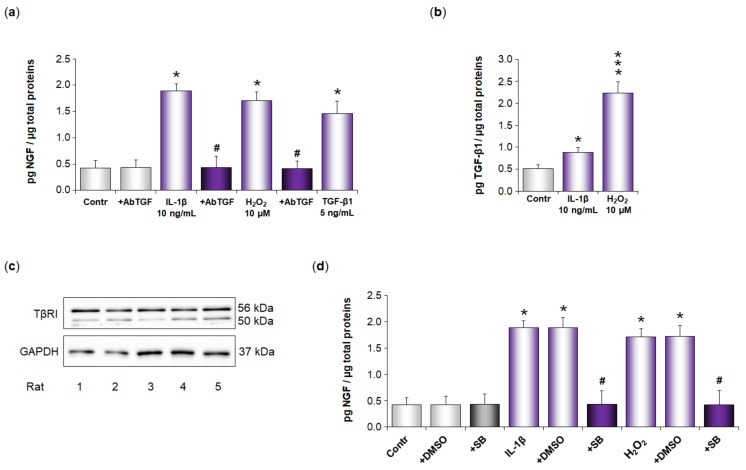
Role of TGF-β1 in IL-1β- or H_2_O_2_-induced increase in NGF secretion by rat whole pulmonary arteries. (**a**) NGF secretion by rat whole pulmonary arteries was assessed in the absence or presence of interleukin-1β (IL-1β, 10 ng/mL, 24 h), hydrogen peroxide (H_2_O_2_, 10 µM, 24 h), or transforming growth factor-β1 (TGF-β1, 5 ng/mL, 24 h). Involvement of TGF-β1 in IL-1β- or H_2_O_2_-induced NGF secretion was investigated through pre-treatment (30 min) of pulmonary arteries with anti-TGF-β1 blocking antibodies (AbTGF, 1 µg/mL). The effect of these antibodies on NGF basal secretion was evaluated. NGF secretion was determined by ELISA (results expressed as pg NGF/µg total proteins). The data represent the means ± SEM with experiments conducted on pulmonary arteries of *n* = 5 rats per group. * *p* < 0.05 versus untreated pulmonary arteries (Contr). ^#^ *p* < 0.05 versus pulmonary arteries treated with either IL-1β or H_2_O_2_. (**b**) TGF-β1 secretion by rat whole pulmonary arteries was assessed in the absence or presence of interleukin-1β (IL-1β, 10 ng/mL, 24 h) or hydrogen peroxide (H_2_O_2_, 10 µM, 24 h). TGF-β1 secretion was determined by ELISA (results expressed as pg TGF-β1/µg total proteins). The data represent the means ± SEM with experiments conducted on pulmonary arteries of *n* = 5 rats per group. * *p* < 0.05 and *** *p* < 0.001 versus untreated pulmonary arteries (Control). (**c**) Basal protein expression of TβRI was assessed by Western blotting analysis in rat whole pulmonary arteries. The immunoblots presented were performed on pulmonary arteries from five different rats (rats 1–5). Results are normalized to glyceraldehyde-3-phosphate dehydrogenase (GAPDH). (**d**) NGF secretion by rat whole pulmonary arteries was assessed in the absence or presence of interleukin-1β (IL-1β, 10 ng/mL, 24 h) or hydrogen peroxide (H_2_O_2_, 10 µM, 24 h). Involvement of TβRI was investigated through pre-treatment (30 min) of pulmonary arteries with SB525334, an inhibitor of TβRI receptor kinase activity (SB, 1 µM). The effect of this inhibitor on NGF basal secretion was investigated. In addition, the effects of dimethyl sulfoxide (DMSO), the vehicle of the SB compound, on NGF basal and induced secretions were evaluated. NGF secretion was determined by ELISA (results expressed as pg NGF/µg total proteins). The data represent the means ± SEM with experiments conducted on pulmonary arteries of *n* = 5 rats per group. * *p* < 0.05 versus untreated pulmonary arteries (Contr). ^#^ *p* < 0.05 versus pulmonary arteries treated with IL-1β or H_2_O_2_.

Finally, we investigated whether IL-1β and H_2_O_2_ also increase NGF secretion in rat whole pulmonary arteries by a similar mechanism. Our results show that NGF pulmonary arterial basal secretion was significantly increased after IL-1β treatment at 10 ng/mL (24 h) (from 0.42 ± 0.14 to 1.89 ± 0.13 pg NGF/µg total proteins, *p* < 0.05, Figure 8a). NGF pulmonary arterial basal secretion was also significantly increased after H_2_O_2_ treatment at 10 µM (24 h) (from 0.42 ± 0.14 to 1.71 ± 0.15 pg/mL of NGF, *p* < 0.05, Figure 8a). IL-1β- or H_2_O_2_-induced increase in NGF pulmonary arterial secretion was totally blocked after treatment with anti-TGF-β1 blocking antibodies (*p* < 0.05), whereas anti-TGF-β1 blocking antibodies did not alter NGF basal secretion (Figure 8a).

In parallel, we also showed that treatment of pulmonary arteries with exogenous TGF-β1 at 5 ng/mL significantly triggered NGF secretion (from 0.42 ± 0.14 to 1.46 ± 0.23 pg NGF/µg total proteins, *p* < 0.05, Figure 8a). TGF-β1 pulmonary arterial basal secretion was significantly increased after IL-1β treatment at 10 ng/mL (from 0.51 ± 0.09 to 0.88 ± 0.11 pg TGF-β1/µg total proteins, *p* < 0.05, Figure 8b). TGF-β1 pulmonary arterial basal secretion was also significantly increased after H_2_O_2_ treatment at 10 µM (from 0.51 ± 0.09 to 2.23 ± 0.26 pg TGF-β1/µg total proteins, *p* < 0.001, Figure 8b).

Finally, we showed that the TGF-β1 receptor TβRI was expressed in rat pulmonary arteries (Figure 8c), and that an IL-1β- or H_2_O_2_-induced increase in NGF secretion was totally blocked with an inhibitor of TβRI kinase activity (SB525334, 1 µM, Figure 8d). Treatment with the inhibitor vehicle alone (DMSO) did not alter NGF neither basal nor induced secretions, and, likewise, treatment with the inhibitor of TβRI kinase activity did not alter NGF pulmonary arterial basal secretion (Figure 8d).

## 4. Discussion

**Figure 9:**
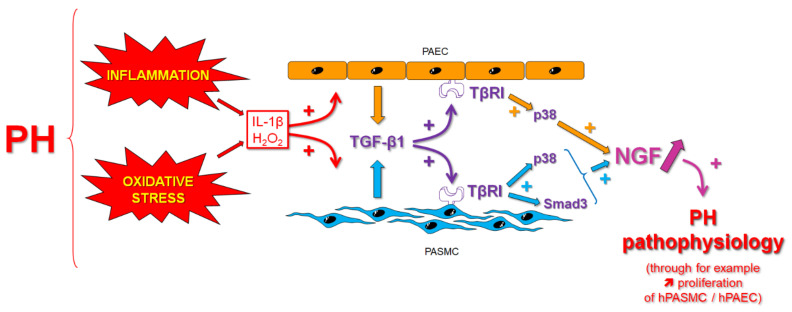
Potential mechanisms of TGF-β1-induced NGF pulmonary arterial secretion in pulmonary hypertension. Inflammation and oxidative stress are pathological conditions in pulmonary hypertension (PH). Increased production of pro-inflammatory cytokines such as interleukin-1β (IL-1β) or of reactive oxygen species such as hydrogen peroxide (H_2_O_2_) can stimulate pulmonary arterial cells such as pulmonary arterial smooth muscle (PASMC) or endothelial cells (PAEC) to secrete transforming growth factor-β1 (TGF-β1). This factor then acts in an autocrine fashion on the same cells through activation of its TβRI receptor and of Smad3 and/or p38-dependent signaling pathways to induce the secretion of nerve growth factor (NGF). This mechanism may contribute to NGF increased pulmonary arterial secretion observed in PH. Since TGF-β1 also plays a pathological role in this disease, such mechanisms of TGF-β1-dependent increase in NGF pulmonary arterial secretion may be relevant to human PH pathophysiology.

In this study, we demonstrate that IL-1β, a pro-inflammatory cytokine, and H_2_O_2_, a non-radical molecule belonging to the reactive oxygen species (ROS), trigger NGF secretion from human pulmonary arterial smooth muscle and endothelial cells. This effect involves secretion of TGF-β1 by these cells, which, in turn, activates its TβRI receptor on the same cells and signaling pathways involving Smad3 and/or p38 to stimulate NGF secretion via autocrine signaling (Figure 9). Similar effects are observed in whole rat pulmonary arteries, suggesting that a TGF-β1-dependent increase in NGF secretion may occur in pulmonary arteries in vivo and thus contribute to NGF increased expression in PH.

We have previously shown that NGF plays a pathophysiological role in PH and that its pulmonary arterial expression is increased in this disease [5]. However, the mechanism contributing to NGF increased expression in PH was still unknown. In this study, we focused on a possible role of inflammation and/or oxidative stress on NGF pulmonary arterial expression.

We demonstrate here that IL-1β, a pro-inflammatory cytokine, and H_2_O_2_, a non-radical ROS, increase NGF pulmonary arterial secretion by hPASMC and hPAEC. Our results also show that IL-1β and H_2_O_2_ increase NGF mRNA and protein expression in both hPASMC and hPAEC, probably leading to NGF-increased secretion by these cells. These results are in accordance with the literature showing the ability of IL-1β to trigger NGF expression/secretion by other lung cell types, such as fibroblasts [19], epithelial cells [20], or bronchial smooth muscle cells [21]. Other pro-inflammatory cytokines, such as, TNF-α can also trigger NGF secretion from these lung cells [19], and accordingly, we observed similar results in both hPASMC and hPAEC (Figure S6). In addition, in accordance with our results, previous studies have shown that oxidative stress and ROS trigger NGF expression from various cell types [24,25,26,27]. Inflammation is a critical PH pathophysiological feature [6,10,11], with increased expression of numerous pro-inflammatory cytokines such as IL-1β [35,36,37]. The source of increased IL-1β in PH may include both infiltrated inflammatory cells [38], such as in particular macrophages [39], but also pulmonary arterial structural cells such as hPASMC and hPAEC [5]. Oxidative stress is also a critical PH pathological feature, with increased ROS production in the pulmonary vasculature, contributing to pulmonary arterial remodeling and altered reactivity [22,23,40]. Our results therefore suggest that, in pulmonary arteries, increased levels of both pro-inflammatory cytokines and ROS may contribute to the increased NGF pulmonary arterial expression evidenced in PH in vivo.

Since (i) chronic hypoxia is responsible for some PH forms, such as in PH associated with chronic obstructive pulmonary diseases (COPD) [41], and (ii) previous data in the literature showed that hypoxia is able to enhance NGF expression in other cell types [42], we also investigated whether hypoxia may alter NGF pulmonary arterial secretion. In our experiments, in the tested conditions (1% O_2_ during 24, 48, or 72 h), hypoxia failed to alter NGF secretion from human pulmonary arterial cells (Figure S7).

We then investigated the mechanism of IL-1β or H_2_O_2_-increased NGF pulmonary arterial secretion. Very interestingly, the present results demonstrate an autocrine signaling of TGF-β1 activating its TβRI receptor and p38 and/or Smad3-dependent signaling pathways in both human pulmonary arterial cells and rat whole pulmonary arteries. In accordance with our results, previous studies have shown TβRI receptor expression in the pulmonary vasculature, particularly in PASMC and PAEC [43,44], and the ability of these cells to express and secrete TGF-β1 [45,46,47]. TGF-β1 is a growth factor involved in PH pathophysiology [31,32,33,34], whose expression is also increased in this disease [28,29,30]. In addition, alterations in TGF-β1-dependent canonical (involving Smad3) and non-canonical (involving p38) signaling pathways have been observed in PH [31,32,33]. In accordance with our results, previous in vitro studies have also shown TGF-β1 secretion triggered in other cell types by IL-1β [48,49] or ROS [50,51]. In addition, in previous studies, TGF-β1 has been shown to increase NGF secretion by other cell types through activation of Smad3 and/or p38-dependent signaling pathways, such as, for example, in chondrocytes [52] or in systemic vascular smooth muscle cells [53]. Moreover, still in accordance with our results, an autocrine role for TGF-β1 has already been described in hPASMC, in which hypoxia triggers TGF-β1 production, with TGF-β1 then acting in an autocrine fashion to promote cell proliferation [46].

Finally, our results show that NGF secretion triggered by IL-1β contributes to IL-1β functional effects, i.e., IL-1β-induced proliferation of hPASMC and hPAEC. Since (i) increased levels of both IL-1β [35] and NGF [5] have been reported in PH, (ii) hPASMC and hPAEC proliferation is a relevant pathophysiological feature contributing to pulmonary arterial remodeling in PH [6], and (iii) both IL-1β [54,55,56,57] and NGF [5,58,59] display proliferative effects on such cell types, our results therefore further support the relevance of the mechanisms evidenced in this study in human PH.

In conclusion, we previously showed that NGF plays a critical role in PH pathophysiology, and a better understanding of the mechanism involved in NGF increased pulmonary arterial expression in PH may help to confirm whether NGF targeting opens new therapeutic perspectives in this disease. In the present study, we show that inflammatory and oxidative stress conditions in PH trigger pulmonary arterial TGF-β1 secretion. The TGF-β1 released may then act in an autocrine manner on pulmonary arterial cells to secrete NGF, thus contributing to NGF increased pulmonary arterial expression and pulmonary arterial remodeling in PH. Our results show similar mechanisms in both human pulmonary arterial cells and rat whole pulmonary arteries. Our results are also in accordance with recent single-cell studies, performed either in PH animal models [60,61] or on pulmonary arterial cells from PH patients [62] and showing altered expression of TGF-β1 and/or TGF-β1 signaling pathways in particular in pulmonary arterial endothelial cells. Since TGF-β1 also plays a pathological role in PH, our results therefore suggest that such a mechanism of TGF-β1-dependent increase in NGF pulmonary arterial secretion may be relevant to human PH pathophysiology.

## Data Availability

Data is contained within the article or Supplementary Materials.

## Supplementary Materials

The following supporting information can be downloaded at: https://www.mdpi.com/article/10.3390/cells11182795/s1: Supplementary material and methods; Table S1: Characteristics of control donors. Figure S1: Characterization of hPASMC and hPAEC phenotypes; Figure S2: Cell viability of pulmonary arterial cells after exposure to IL-1β or H_2_O_2_; Figure S3: NGF mRNA expression by human pulmonary arterial cells after treatment with IL-1β or H_2_O_2_; Figure S4: NGF protein expression by human pulmonary arterial cells after treatment with IL-1β or H_2_O_2_; Figure S5: TGF-β1 secretion triggered by IL-1β or H_2_O_2_ in human pulmonary arterial cells after pre-treatment with anti-NGF blocking antibodies; Figure S6: NGF secretion by human pulmonary arterial cells after treatment with TNF-α; Figure S7: NGF secretion by human pulmonary arterial cells placed under hypoxic conditions.
