# Supplementary material for: Inflammation and Oxidative Stress Induce NGF Secretion by Pulmonary Arterial Cells through a TGF-β1-Dependent Mechanism
Source: Cells. 2022 Sep 7;11(18):2795. doi: 10.3390/cells11182795 (PMC9496672; doi:10.3390/cells11182795)
Supplement: Supplementary file 1 [file cells-11-02795-s001.zip › cells-1681768-supplementary.pdf]

Online Supplement

Inflammation and oxidative stress induce NGF secretion by pulmonary arterial cells through a TGF-β1-dependent mechanism

Clément Bouchet[1,2], Guillaume Cardouat[1,2], Matthieu Douard[1,2,3], Florence Coste[4], Paul Robillard[1,2], Frédéric Delcambre[5], Thomas Ducret[1,2], Jean-François Quignard[1,2], Pierre Vacher[1,2], Isabelle Baudrimont[1,2], Roger Marthan[1,2,5], Patrick Berger[1,2,5], Christelle Guibert[1,2#], Véronique Freund-Michel[1,2#*]

[#] *These authors contributed equally as last authors*

[1] *Centre de Recherche Cardio-Thoracique de Bordeaux, Univ. Bordeaux, U1045, 33600 Pessac, France*

[2] *INSERM (Institut National de la Santé Et de la Recherche Médicale), Centre de Recherche Cardio-Thoracique de Bordeaux, U1045, 33600 Pessac, France*

[3] *IHU Institut de Rythmologie et Modélisation Cardiaque (LIRYC), 33600 Pessac, France*

[4] *Laboratoire de Pharm-écologie Cardiovasculaire (LaPEC-EA 4278), Université d'Avignon et des Pays du Vaucluse, 84000 Avignon, France*

[5] *CHU de Bordeaux, 33000 Bordeaux, France*

***<u>Corresponding author</u>**

Véronique FREUND-MICHEL

Centre de Recherche Cardio-Thoracique de Bordeaux, INSERM U1045

Plateforme Technologique d'Innovation Biomédicale, Hôpital Xavier Arnozan

Avenue du Haut Lévêque, 33604 Pessac Cedex, France

Tel: + 33 5 47 30 27 50 / +33 5 47 30 27 51 / + 33 5 57 57 45 70

E-mail: veronique.michel@u-bordeaux.fr

Supplementary material and methods

Control donors

Human lung tissues were obtained from 20 patients undergoing resection for pulmonary carcinoma, either non-smokers or smokers, but without any other associated pathology. Available characteristics of these patients are shown in Table S1a. All subjects were recruited from the Centre Hospitalier Universitaire of Bordeaux and gave their written informed consent to participate in the study after the nature of the procedure had been fully explained. The study followed recommendations outlined in the Helsinki Declaration and received approval from the local ethics committee (Comité de Protection des Personnes, Sud-Ouest et Outre-mer III, Bordeaux, France).

The lung specimens were collected at distance from tumor foci, and immediately transferred to the laboratory in sterile DMEM (Sigma Aldrich, Saint-Quentin-Fallavier, France). From a macroscopically part of each of the specimens, segments of pulmonary arteries (3rd to 4th division) were carefully dissected under a dissecting microscope. After removal of surrounding tissues, associated bronchi and adventitia, pulmonary arterial segments were opened and the inner side was subjected to enzymatical dissociation. After this procedure, the segments' inner side was scraped to collect hPAEC, and the remaining tissue, i.e. the pulmonary arterial smooth muscle layer, was cut into small pieces measuring 1-2 mm² to be cultured. Further details regarding cell isolation, culture and characterization are provided in the next paragraph.

Isolation, culture and characterization of human pulmonary arterial endothelial (hPAEC) and smooth muscle cells (hPASMC)

Once the dissected pulmonary arterial rings were opened, their inner face was subjected to collagenase type I digestion (40 U/mL, 30 min, 37°C, ThermoFisher Scientific, Illkirch, France) and then scraped to collect hPAEC. The obtained cell suspension was centrifuged (250*x g*, 5 min, 4°C) and cells were then suspended in Endothelial Cell Growth Medium 2 (Promocell, Heidelberg, Germany) and plated onto 0.1% fibronectin-coated wells (fibronectin from Sigma-Aldrich, Saint-Quentin-Fallavier, France). Immunomagnetic purification of hPAEC was then performed with the CD31 MicroBead Kit, Human (Miltenyi Biotec, Paris, France). Cells were then immunocytochemically characterized as endothelial cells using monoclonal antibodies against CD31 and von Willebrand Factor (vWF) (both antibodies from Sigma-Aldrich) (Figure S1a). Cells positive for CD31 and vWF constituted approximately 90 % of our cultured cells. Cells were then cultured using the Endothelial Cell Basal Medium (Promocell), supplemented with growth factors for cell

culture and the DetachKit® for trypsinization (Promocell), and used for the study between passages 3 and 6. Some experiments were also conducted on hPAEC obtained commercially (Promocell). Available characteristics of the donors for these cells provided by the supplier are shown in Table S1b.

After digestion of the intimal layer and once hPAEC had been scraped, the remaining pulmonary arterial smooth muscle layer was cut into small pieces (1-2 mm^2) and 5 to 6 explants were then transferred into each well of a 6-well plate. Tissues were incubated with DMEM supplemented with 10% fetal calf serum (ThermoFisher Scientific), penicillin (100 units/mL) and streptomycin (100 µg/mL) (both from Sigma-Aldrich) at 37 °C and 5% CO_2. After 2 weeks of incubation, cells were passaged and transferred in new cell-culture flasks. Cells were characterized as smooth muscle cells both morphologically (typical "hills and valleys" morphology) and immunocytochemically using monoclonal antibodies against α-smooth muscle actin, vimentin and calponin (all antibodies from Sigma-Aldrich) (Figure S1b). Cells positive for α-smooth muscle actin, vimentin and calponin constituted approximately 95 % of our cultured cells. Cells were then cultured in the same conditions and used for the study between passages 2 and 6.

Supplementary table

TABLE S1. Characteristics of control donors

a) Human lung tissues were obtained from 20 patients undergoing resection for pulmonary carcinoma, either non-smokers or smokers, but without any other associated pathology.

Controls	Age (years)	Sex	Smoking status	Cells isolated
1	54	M	S	PASMC
2	72	F	S	PASMC
3	49	F	NS	PASMC
4	54	M	S	PASMC
5	69	M	S	PASMC
6	73	F	S	PAEC
7	66	M	NS	PAEC
8	60	F	S	PASMC
9	72	M	S	PASMC
10	44	F	S	PASMC
11	79	F	NS	PASMC
12	72	M	NS	PASMC
13	59	M	NS	PASMC
14	18	M	NS	PASMC
15	69	F	NS	PASMC
16	64	F	NS	PAEC
17	55	F	NS	PASMC
18	23	M	NS	PASMC
19	64	F	S	PAEC
20	69	F	NS	PASMC + PAEC

b) Some experiments were also conducted on hPAEC obtained commercially (Promocell). Available characteristics of the donors for these cells provided by the supplier are:

Controls	*Age (years)*	*Sex*	*Smoking status*
C1	*49*	*M*	*S*
C2	*56*	*F*	*S*
C3	*45*	*M*	*NS*
C4	*51*	*F*	*NS*

F: female; M: male; S: smoker; NS: non-smoker; PASMC: pulmonary arterial smooth muscle cells; PAEC: pulmonary arterial endothelial cells.

Supplementary figures

Figure S1: Characterization of hPASMC and hPAEC phenotypes

Figure S2: Cell viability of pulmonary arterial cells after exposure to IL-1β or H_2O_2

Figure S3: NGF mRNA expression by human pulmonary arterial cells after treatment with IL-1β or H_2O_2

Figure S4: NGF protein expression by human pulmonary arterial cells after treatment with IL-1β or H_2O_2

Figure S5: TGF-β1 secretion triggered by IL-1β or H_2O_2 in human pulmonary arterial cells after pre-treatment with anti-NGF blocking antibodies

Figure S6: NGF secretion by human pulmonary arterial cells after treatment with TNF-α

Figure S7: NGF secretion by human pulmonary arterial cells placed under hypoxic conditions

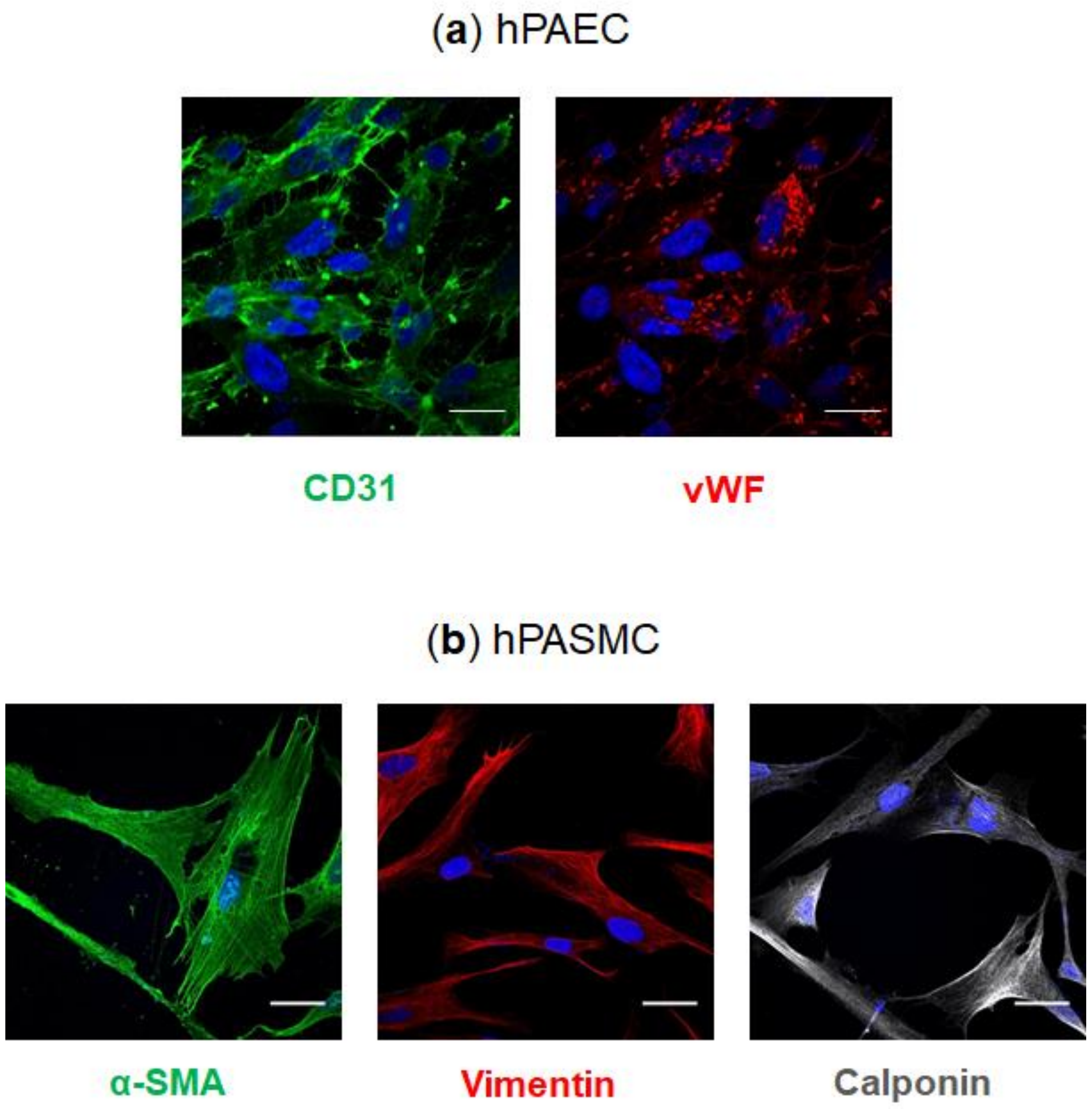

FIGURE S1. Characterization of hPAEC and hPASMC phenotypes

Representative confocal immunofluorescence fields of human pulmonary arterial endothelial cells (hPAEC, a) or human pulmonary arterial smooth muscle cells (hPASMC, b) from control donors. hPAEC were stained with a primary antibody against CD31 (green) or Von Willebrand factor (vWF, red), recognized with an Alexa-Fluor 488-conjugated secondary antibody. hPASMC were stained with a primary antibody against α-smooth muscle actin (α-SMA, green), vimentin (red) or calponin (white), recognized with an Alexa-Fluor 488-conjugated secondary antibody. Cell nuclei were stained with DAPI (blue). All images were taken with the same illumination time, and are representative of cells from all control donors. Scale bar represents 25 µm for hPAEC and 100 µm for hPASMC.

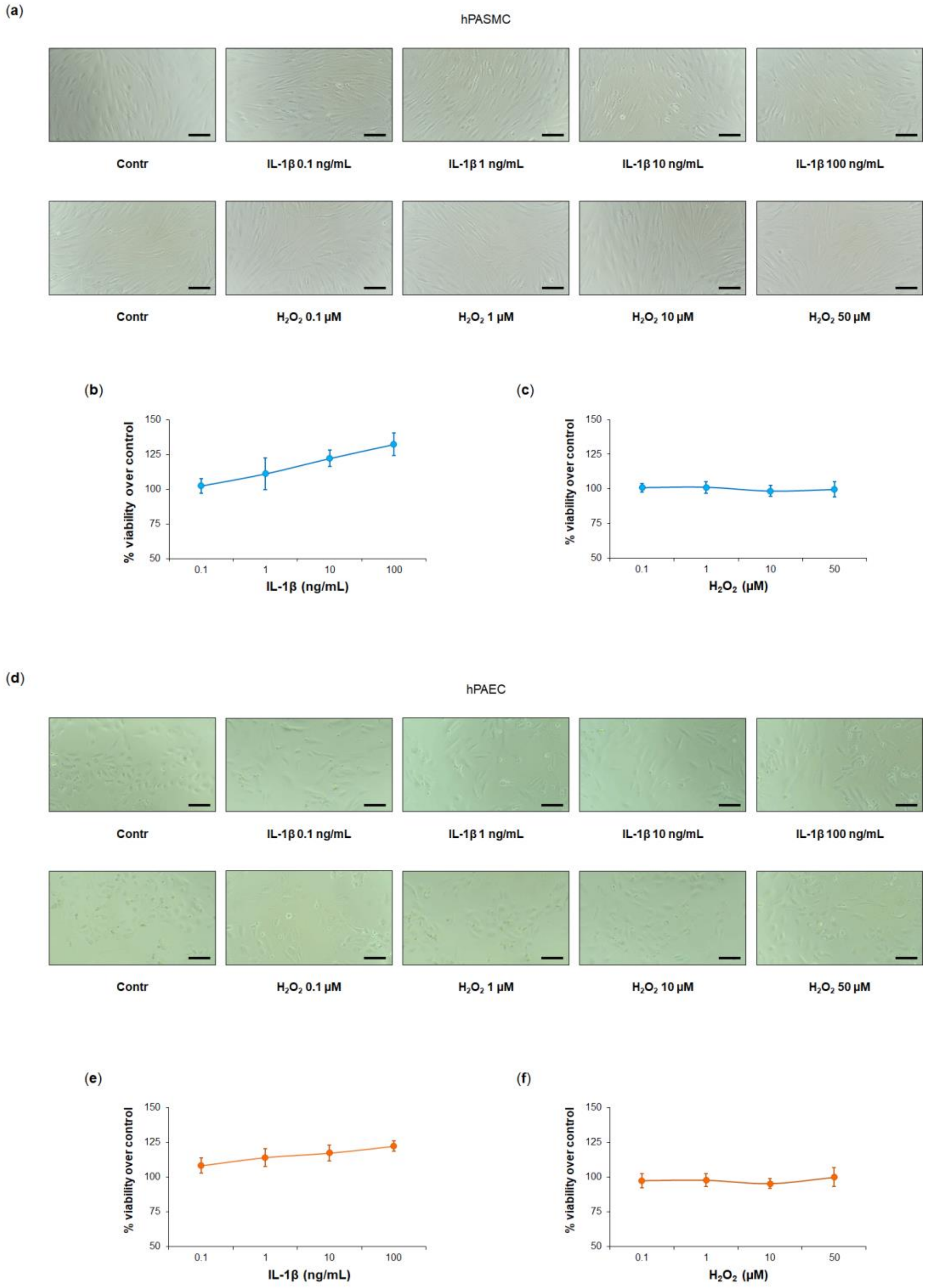

FIGURE S2. **Cell viability of pulmonary arterial cells after exposure to IL-1β or H_2O_2**

Viability of human pulmonary arterial smooth muscle cells (hPASMC, a-c) or of human pulmonary arterial endothelial cells (hPAEC, d-f) was assessed in the absence or presence of interleukin-1β (IL-1β, 0.1-100 ng/mL, 24 h, a, b, d, e) or of hydrogen peroxide (H_2O_2, 0-50 µM, 24 h, a, c, d, f). Cell viability was measured by the colorimetric WST-1 assay. Results are expressed as the percentage of viability of treated cells, as compared to control cells (100 %). The data represent the means ± SD of n = 3 independent experiments performed in triplicate on cells from three control donors.

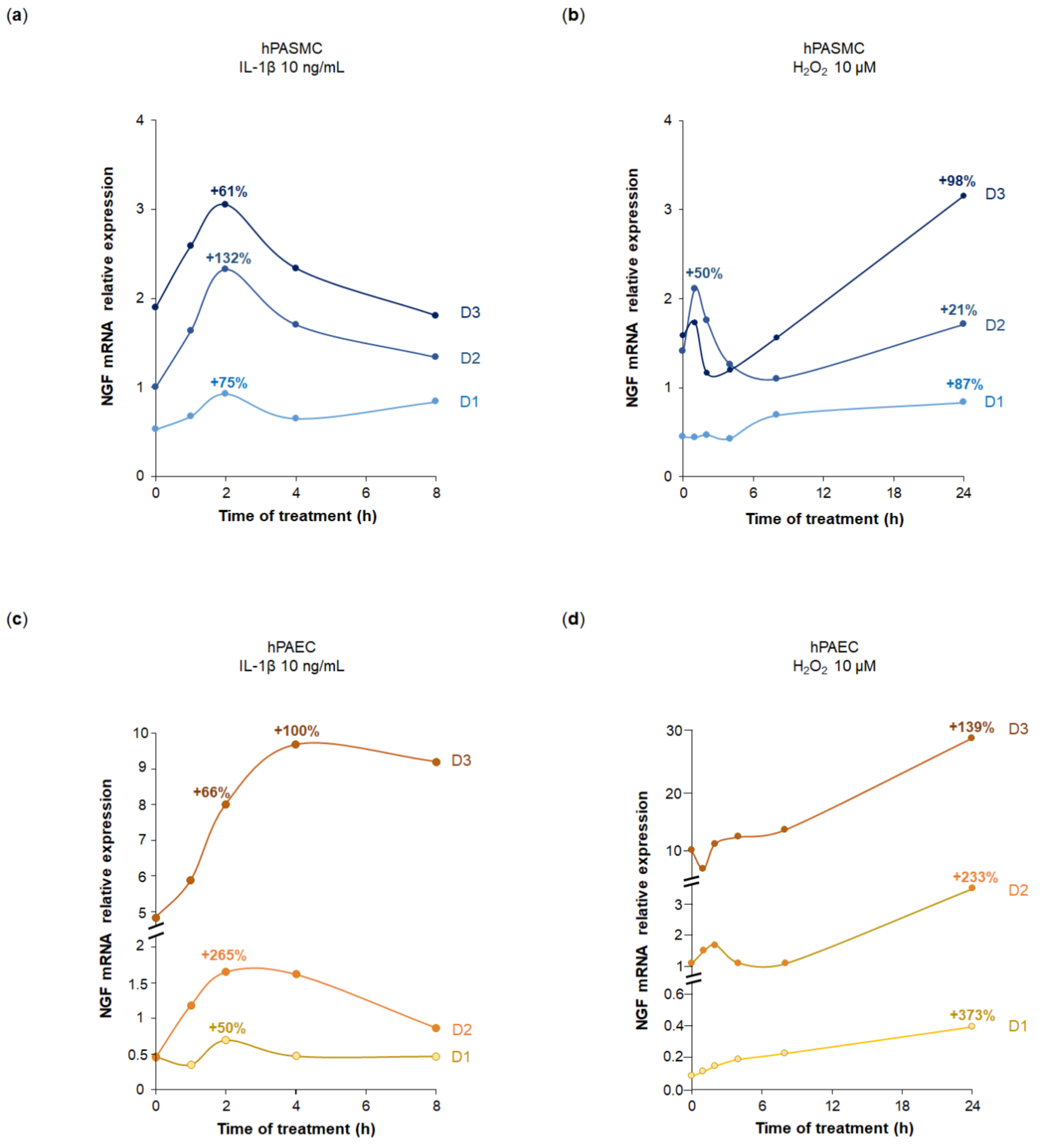

FIGURE S3. NGF mRNA expression by human pulmonary arterial cells after treatment with IL-1β or H_2O_2

NGF mRNA expression by human pulmonary arterial smooth muscle cells (hPASMC, a and b) or by human pulmonary arterial endothelial cells (hPAEC, c and d) was assessed in the absence or presence of interleukin-1β (IL-1β, 10 ng/mL, 0 to 8 h, a and c) or of hydrogen peroxide (H_2O_2, 10 µM, 0 to 24 h, b and d). NGF mRNA expression was determined by quantitative real-time PCR (results expressed as NGF mRNA relative expression using the comparative $2^{-\Delta\Delta Ct}$ method, and normalized to the mRNA expression level of 2 or 3 internal housekeeping genes). The data represent raw data of n = 3 independent experiments performed in duplicate on cells from three control donors (D1, D2 or D3). The percentages on the graphs indicate the percentage increase in NGF mRNA expression for each donor at the time pointed out as compared to control cells at time 0 h.

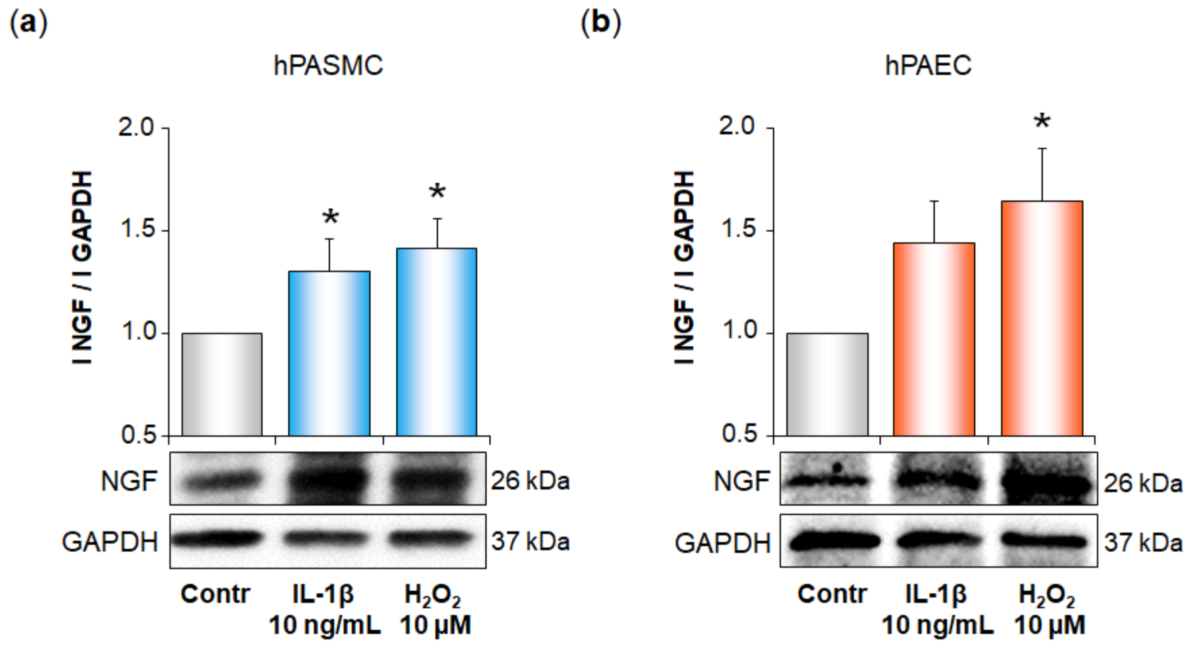

FIGURE S4. **NGF protein expression by human pulmonary arterial cells after treatment with IL-1β or H_2O_2**

NGF protein expression by human pulmonary arterial smooth muscle cells (hPASMC, a) or by human pulmonary arterial endothelial cells (hPAEC, b) was assessed in the absence or presence of interleukin-1β (IL-1β, 10 ng/mL, 24 h) or of hydrogen peroxide (H_2O_2, 10 µM, 24 h). NGF protein expression was determined by Western blotting analysis. Immunoblots presented are representative of experiments conducted on pulmonary arterial cells from n=7-8 control donors, showing identical results. Quantification of NGF protein expression is shown in the bar graphs, with the data expressed as a fold-increase in NGF protein expression compared to untreated control cells and presented as means ± SEM. Results are normalized to glyceraldehyde-3-phosphate dehydrogenase (GAPDH). *: $p<0.05$ *versus* untreated control cells.

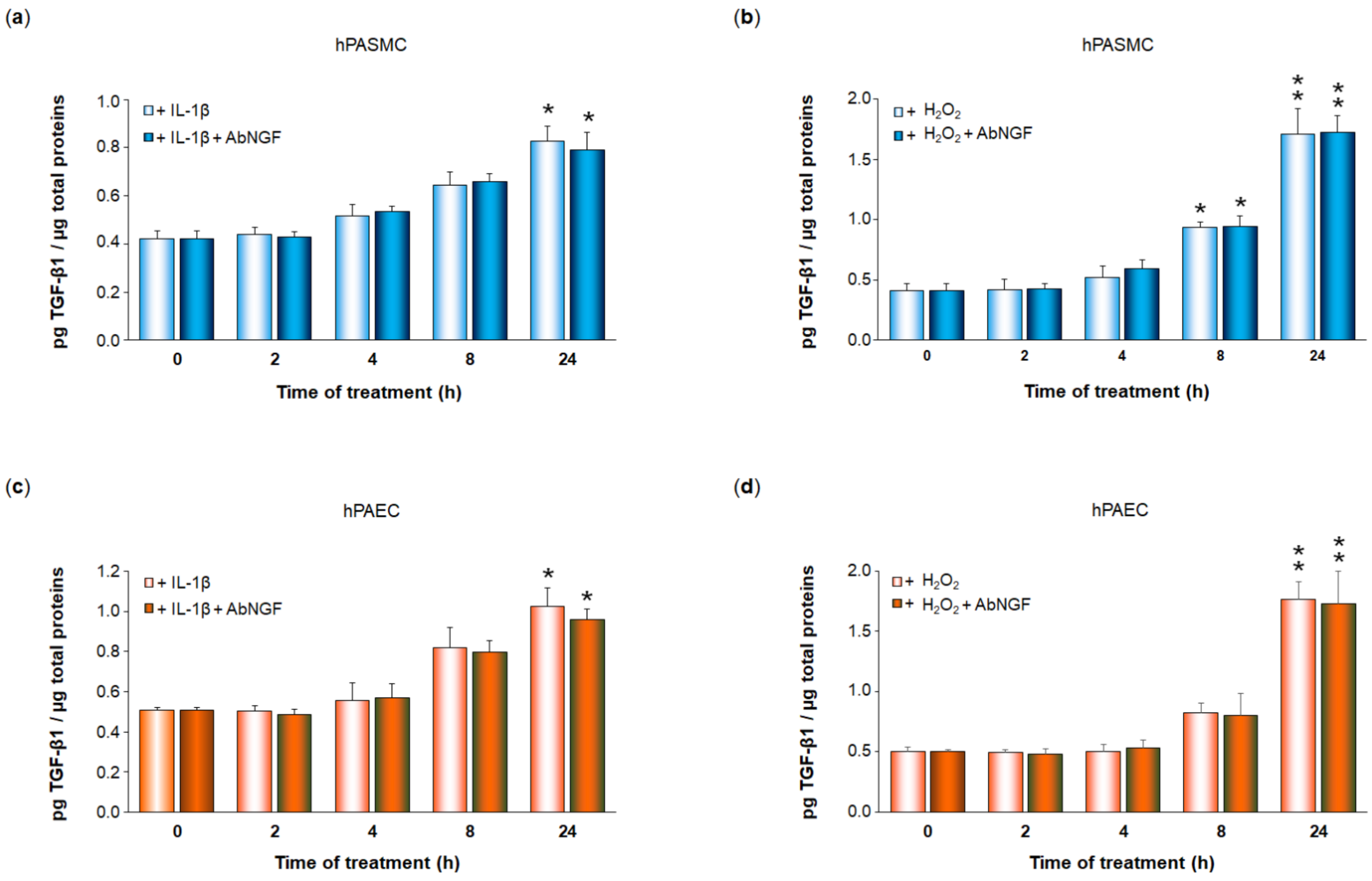

FIGURE S5. **TGF-β1 secretion triggered by IL-1β or H_2O_2 in human pulmonary arterial cells after pre-treatment with anti-NGF blocking antibodies**

TGF-β1 secretion by human pulmonary arterial smooth muscle cells (hPASMC, a and b) or human pulmonary arterial endothelial cells (hPAEC, c and d) was assessed in the absence or presence of interleukin 1β (IL-1β, 10 ng/mL, 0 to 24 h, a and c) or of hydrogen peroxide (H_2O_2, 10 µM, 0 to 24 h, b and d). Involvement of NGF in TGF-β1 secretion was investigated through pre-treatment (45 min) of hPASMC (a and b) or hPAEC (c and d) with anti-NGF blocking antibodies (AbNGF, 1 µg/mL). TGF-β1 secretion was determined by ELISA (results expressed as pg TGF- β1 / µg total proteins). The data represent the means ± SEM of n = 3-4 independent experiments performed in duplicate on cells from three or four control donors. *: $p<0.05$ and **: $p<0.01$ *versus* untreated control cells (cells at 0 h).

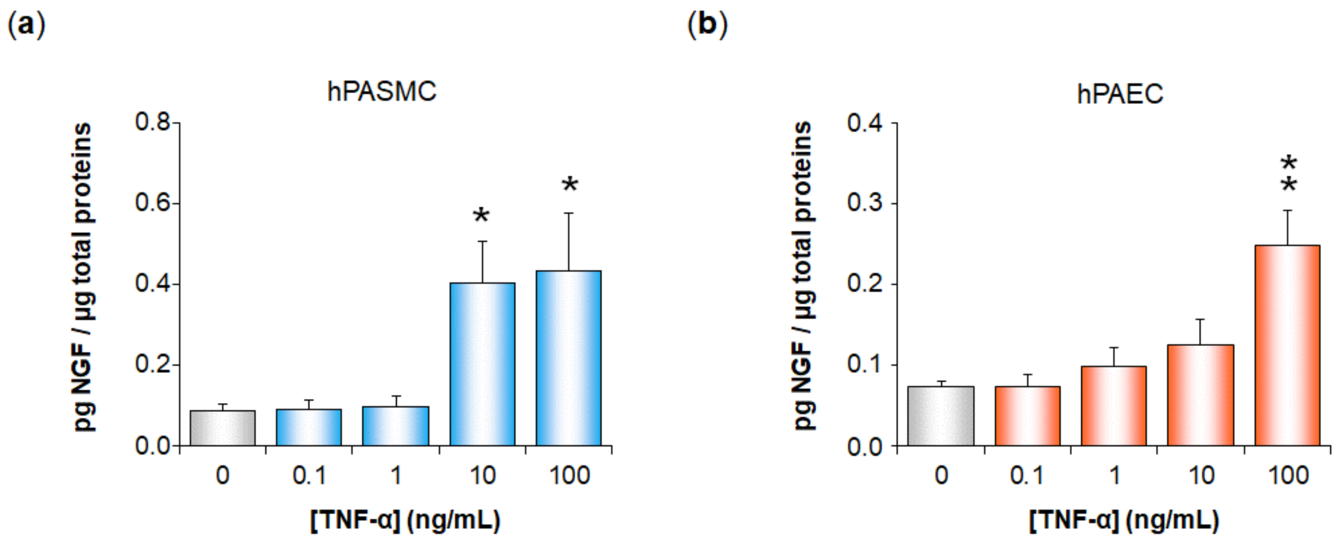

<u>FIGURE S6. NGF secretion by human pulmonary arterial cells after treatment with TNF-α</u>

NGF secretion by human pulmonary arterial smooth muscle (hPASMC, a) or endothelial cells (hPAEC, b) was assessed in the absence or presence of tumor necrosis factor-α (TNF-α, 0.1-100 ng/mL, 24 h). NGF secretion was determined by ELISA (results expressed as pg NGF / µg total proteins). The data represent the means ± SEM of n = 5 independent experiments performed in triplicate on cells from three to five control donors. *: $p<0.05$ and **: $p<0.01$ *versus* untreated control cells.

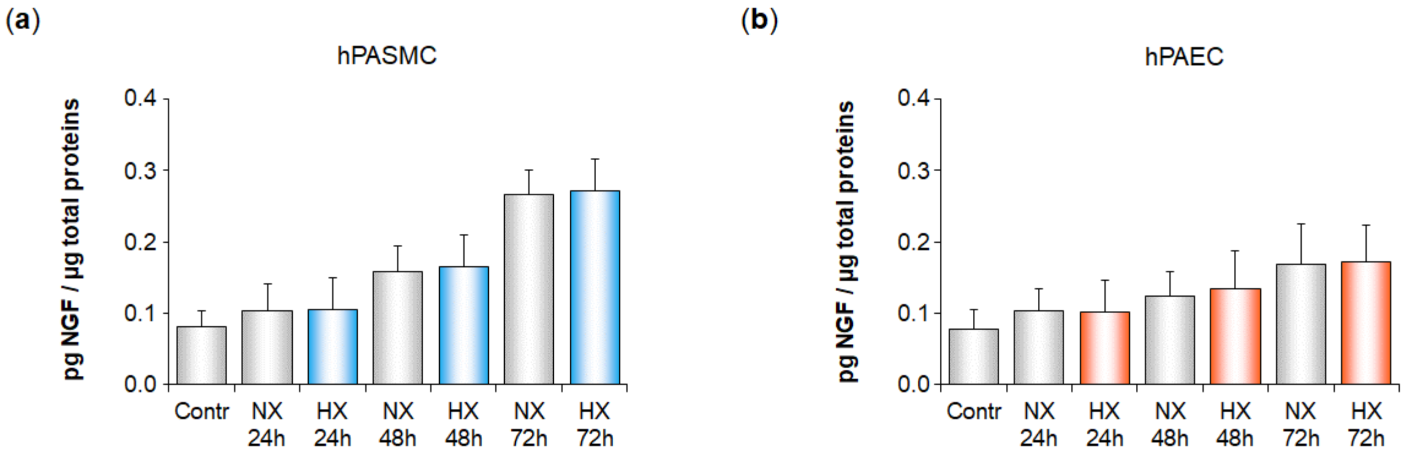

<u>FIGURE S7. NGF secretion by human pulmonary arterial cells placed under hypoxic conditions</u>

NGF secretion by human pulmonary arterial smooth muscle (hPASMC, a) or endothelial cells (hPAEC, b) was assessed after 0, 24, 48 or 72h, after exposure to either normoxic (NX) or hypoxic conditions (HX, 1% O_2). NGF secretion was determined by ELISA (results expressed as pg NGF / µg total proteins). The data represent the means ± SEM of n = 5 independent experiments performed in triplicate on cells from three to five donors.
